# Predictive factors in patients with EGFR mutation-negative non-small cell lung cancer treated with erlotinib

**DOI:** 10.3892/ol.2014.2548

**Published:** 2014-09-18

**Authors:** HIDENOBU ISHII, KOICHI AZUMA, KAZUHIKO YAMADA, TAKASHI KINOSHITA, YOUHEI IMAMURA, TOMOAKI HOSHINO

**Affiliations:** Division of Respirology, Neurology and Rheumatology, Department of Internal Medicine, Kurume University School of Medicine, Kurume, Fukuoka 830-0011, Japan

**Keywords:** pulmonary metastasis, non-small cell lung cancer, erlotinib, epidermal growth factor receptor mutation

## Abstract

Factors predicting the efficacy of erlotinib treatment in patients with EGFR mutation-negative non-small-cell lung cancer (NSCLC) have not been well studied. This retrospective study investigates whether patient characteristics, such as site of metastasis, can predict the efficacy of erlotinib treatment in NSCLC patients. In total, 53 EGFR mutation-negative NSCLC patients treated with erlotinib were enrolled, and the associations between clinicopathological characteristics and patient survival were analyzed. The EGFR mutation status was determined using the peptide nucleic acid-locked nucleic acid polymerase chain reaction clamp method. Survival curves were obtained using the Kaplan-Meier method. Among the NSCLC patients treated with erlotinib, 27 patients with pulmonary metastasis exhibited significantly longer progression-free survival (PFS) and overall survival (OS) times than those without pulmonary metastasis (median PFS time, 2.9 versus 1.2 months; P=0.0010 and median OS time, 12.4 versus 4.1 months; P=0.0007). Multivariate analyses also revealed that pulmonary metastasis independently correlated with PFS and OS times (hazard ratio, 0.39; P=0.0055 and hazard ratio, 0.33; P=0.0022, respectively). Patients with pulmonary metastasis exhibited significantly longer PFS and OS times than those without pulmonary metastasis. The presence of pulmonary metastasis may be a predictive factor in patients with EGFR mutation-negative NSCLC treated with erlotinib.

## Introduction

Non-small-cell lung cancer (NSCLC) is the leading cause of cancer mortality worldwide (1). Recently, molecular-targeting therapies such as gefitinib and erlotinib have gained attention due to their potential to improve survival and reduce toxic side effects in patients with NSCLC (2–4). Four phase III trials with gefitinib or erlotinib in patients with epidermal growth factor receptor (EGFR) mutation-positive NSCLC have demonstrated higher response rates and longer progression-free survival (PFS) times than those of patients who received platinum doublets as first-line chemotherapy (5–8). These results indicate that treatment with EGFR-tyrosine kinase inhibitors (TKIs) may now be the standard treatment for EGFR mutation-positive NSCLC patients. However, the clinical role of EGFR-TKI treatment in EGFR mutation-negative patients has not yet been elucidated. A number of researchers have reported that erlotinib may also have efficacy against EGFR-negative NSCLC (9–11).

The factors predicting the efficacy of erlotinib treatment in patients with EGFR mutation-negative NSCLC have not been well studied. In order to improve the survival of patients with EGFR mutation-negative NSCLC receiving EGFR-TKIs including erlotinib, a biomarker that can predict the efficacy of EGFR-TKIs is required.

The presence of pulmonary metastasis and malignant pleural effusion in patients with NSCLC has also been reported to be a predictive factor of EGFR mutations (12,13). However, the association between these characteristics and the efficacy of erlotinib treatment in patients with EGFR mutation-negative NSCLC remains uncertain. These findings prompted the investigation of the correlation between the efficacy of erlotinib treatment and sites of metastasis in patients with EGFR mutation-negative NSCLC in the current study. It was investigated whether metastasis to specific organs, including pulmonary metastasis and malignant pleural effusion, may predict the efficacy and outcome of erlotinib treatment in patients with EGFR mutation-negative NSCLC.

## Patients and methods

### Patient characteristics

This retrospective study included cases of histologically or cytologically diagnosed NSCLC, which were advanced stage IIIB or IV, according to the International Association for the Study of Lung Cancer staging system (14) or recurrent at initial diagnosis. In total, 206 NSCLC patients were treated with EGFR-TKIs at Kurume University Hospital (Kurume, Japan) between April 2008 and September 2012. Of these patients, 53 were identified as EGFR mutation-negative and thus, were enrolled in this study. The clinical characteristics of the patients, including age, gender, smoking history, tumor histology, Eastern Cooperative Oncology Group (ECOG) performance status (PS) (15), onset of skin rash following treatment and metastatic sites, were recorded. Tumor nodules in the primary (T3) and in other ipsilateral lobes (T4) were included as pulmonary metastases. Tumor response was examined by computed tomography and evaluated using the Response Evaluation Criteria for Solid Tumors, version 1.0 (RECIST, v 1.0) (16). The present study was conducted in accordance with the Declaration of Helsinki and was approved by the Institutional Review Board of Kurume University Hospital (Kurume, Japan).

### DNA extraction and peptic nucleic acid-locked nucleic acid (PNA-LNA) polymerase chain reaction (PCR) clamp assay

For *EGFR* mutation analysis, the PNA-LNA PCR clamp method was adopted, using protocols described previously (17). Specific PNA-LNA probe sets for two mutation sites, exon 19 (delE746-A750) and exon 21 (L858R), were developed and these covered >90% of *EGFR* mutations reported previously in Japan. In brief, the genomic DNA was purified from paraffin-embedded tissues using a QIAamp DNA Micro kit (Qiagen, Valencia, CA, USA). The PCR primers employed were synthesized by Invitrogen Life Technologies (Carlsbad, CA, USA), PNA clamp primers and LNA mutant probes were purchased from FASMEC (Kanagawa, Japan) and Integrated DNA Technologies, Inc., (Coralville, IA, USA), respectively. The PNA-LNA PCR clamp assay was performed using a SDS-7500 System (Applied Biosystems Life Technologies, Foster City, CA, USA).

### Statistical analysis

Fisher’s exact test was used to analyze the significance of associations between patient characteristics and overall response [complete response (CR) and partial response (PR) by RECIST]. The objective response rate (RR) was defined as the proportion of CR or PR. PFS was defined as the period from the date of initiation of erlotinib treatment to the onset of disease progression or mortality from any cause. Overall survival (OS) was measured from the administration of the initial dose of erlotinib until the date of mortality or loss to follow-up. The Kaplan-Meier method was used to assess the survival curves and the log-rank test was used to evaluate the significance of differences between the two groups. The univariate survival analyses were conducted by means of log-rank test, and the multivariate regression was performed using the Cox proportional-hazards regression model. All variables that had P-values of <0.05 were included in the Cox regression model. All tests were two-sided, and P<0.05 was considered to indicate a statistically significant difference. All statistical analyses were conducted using JMP, version 10 (SAS Institute Inc., Cary, NC, USA).

## Results

### Patient characteristics

The clinical characteristics of the 53 patients are shown in Table I. Overall, 13 patients were female and 12 were never-smokers; the age range was 35–80 years (median, 64.2 years). In total, 36 patients had adenocarcinoma and 11 had squamous cell carcinoma. The PS was good (ECOG, 0–1) in 44 patients, and poor (ECOG, 2–3) in the remaining nine patients. Erlotinib was used as the first-line therapy in one patient, as a second-line therapy in 13 patients, as a third-line therapy in 29 patients, and as a fourth-line therapy or thereafter in 10 patients. Among the 53 patients who exhibited distant metastasis, 27 (50.9%), 13 (24.5%), 11 (20.8%), 10 (18.9%), 5 (9.4%), 6 (11.3%) and 14 (26.4%) also had pulmonary, brain, bone, extrathoracic lymph node, adrenal gland and liver metastasis, and malignant pleural effusion, respectively.

### Survival analysis

**Figure 1:**
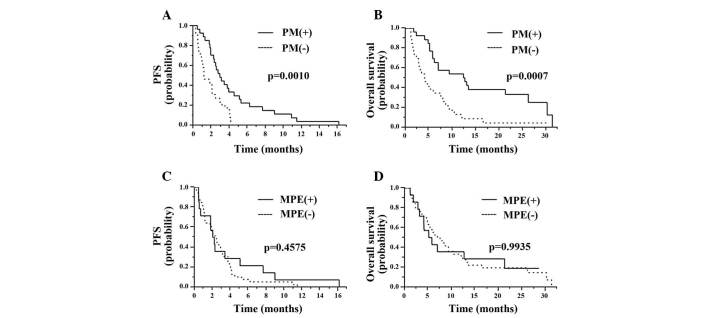
Kaplan-Meier survival curves of (A) PFS and (B) OS according to PM. Kaplan-Meier survival curves of (C) PFS and (D) OS according to MPE. PFS, progression-free survival; OS, overall survival; PM, pulmonary metastasis; MPE, malignant pleural effusion.

In total, four patients responded to erlotinib therapy, exhibiting a response rate of 7.5%. All four of these patients also had pulmonary metastasis and malignant pleural effusion with adenocarcinoma. At the time of analysis, the median duration of follow-up was 9.8 months (range, 1.2–31.3 months). The median PFS time for the patients overall was 2.2 months and the median OS time was 6.2 months. Table II shows the patient demographics, excluding metastatic sites, associated with RR, PFS and OS. The patients with improved PS and skin rash following treatment, exhibited longer PFS and OS times than those with poor PS and without skin rash, as indicated in previous studies (PFS, P=0.0002 and P=0.0077; OS, P<0.0001 and P=0.0026, respectively) (18,19). However, other factors were demonstrated to be unrelated to PFS and OS. The median PFS and median OS times for patients according to metastatic sites are shown in Table III. The PFS and OS did not depend on the presence or absence of extrathoracic lymph node and adrenal gland metastasis. In patients with brain, bone and liver metastasis, the median PFS times were shorter than for those patients without these metastases. Furthermore, patients with liver metastasis exhibited a shorter OS time than patients without liver metastasis. The median PFS times in the two groups of patients with and without pulmonary metastasis were 2.9 months (95% CI, 1.9–4.5 months) and 1.2 months (95% CI, 0.8–2.1 months), respectively (P=0.001; Fig. 1A). Although no significant differences were identified between the response rate in patients with and without pulmonary metastasis, the response rate tended to be higher in patients with pulmonary metastasis (response rate, 14.8 vs. 0.0%; P=0.1110). The median duration of OS in the two groups of patients with and without pulmonary metastasis was 12.4 months (95% CI, 5.8–26.2 months) and 4.1 months (95% CI, 2.3–7.6 months), respectively (P=0.0007; Fig. 1B). The response rate in patients with malignant pleural effusion was significantly higher than that of patients without malignant pleural effusion (response rate, 28.6% vs. 0.0%; P=0.0034). However, as shown in Fig. 1C, the median PFS times in the patients with and without malignant pleural effusion were 2.1 and 2.5 months, respectively (P=0.4575). Furthermore, no significant differences were identified in OS between the patients with and without malignant pleural effusion (median OS time, 5.5 months vs. 7.3 months; P=0.9935; Fig. 1D). Of the 13 variables assessed, six were observed to be significantly associated with PFS in univariate analysis: Pulmonary, brain, bone and liver metastasis, plus the onset of skin rash and PS. The multivariate analyses of PFS demonstrated that pulmonary metastasis was an independent and significant predictive factor for PFS (P=0.0055) (Table IV). By contrast, liver metastasis and poor PS were risk factors for an unfavorable PFS following erlotinib therapy (P=0.0279 and P=0.0214, respectively). Additionally, four factors were observed to be significantly associated with OS in the univariate analysis: Pulmonary and liver metastasis plus the onset of skin rash and PS. The presence of pulmonary metastasis was also an independent and significant prognostic factor in the multivariate analysis (P=0.0022).

## Discussion

This study demonstrated that the presence of pulmonary metastasis was a predictive marker of the outcome in patients with EGFR-negative NSCLC, receiving erlotinib treatment. Previously, a randomized controlled trial (BR21) investigating the effects of erlotinib versus placebo demonstrated that erlotinib significantly prolonged the median OS, PFS and improved the RR in comparison with the placebo (9). Furthermore, subset analysis in this trial demonstrated that erlotinib treatment was effective in patients with EGFR mutation-negative NSCLC. Several studies have reported that skin rashes following erlotinib treatment tend to correlate with the therapeutic efficacy in patients with NSCLC (18,19). Therefore, the requirement for biomarkers that can predict the efficacy of erlotinib therapy prior to initiation is evident. A number of authors have examined the association between the efficacy of EGFR-TKIs and patient demographics, including gender, tumor histology, smoking history and ECOG-PS. However, few studies have evaluated the efficacy of EGFR-TKIs focusing on metastatic sites as a tumor property. The present study investigated the association between patient characteristics, including metastatic sites and the efficacy of erlotinib treatment in EGFR-mutation negative NSCLC, and demonstrated that pulmonary metastasis was a significant and independent factor associated with PFS and OS. Together, these findings suggest that the presence of pulmonary metastasis may be useful for predicting the efficacy of erlotinib in patients with EGFR mutation-negative NSCLC.

Somatic mutations in the *EGFR* gene have been identified as a major determinant of the clinical response to treatment with EGFR-TKIs, such as gefitinib and erlotinib, in individuals with NSCLC (2,3). In the current study, the four patients who responded to erlotinib treatment had pulmonary metastasis and malignant pleural effusion with adenocarcinoma. Recent studies have suggested that the presence of pulmonary metastasis and malignant pleural effusion is predictive of EGFR mutations, as is the case in adenocarcinoma (12,13). In the current study, a number of cases were reanalyzed for EGFR mutations, including minor mutations, such as exon 20 insertions and G719X in exon 18; however, no EGFR mutations were identified in the reanalyzed samples (results not shown), suggesting that erlotinib may be effective in certain patients with EGFR mutation-negative NSCLC. Erlotinib inhibits the activity of EGFR mutation-negative NSCLC tumor cells at a 50% inhibitory concentration of 2–20 nmol/l. By contrast, three-fold higher concentrations of gefitinib are required in order to block mutation-negative EGFR signaling (20,21). In EGFR mutation-negative NSCLC, it is postulated that erlotinib may bind to the EGFR more readily than gefitinib. These results suggest that erlotinib treatment may be effective in patients with EGFR mutation-negative NSCLC. Patients who responded to erlotinib treatment in the current study exhibited a rapid reduction of tumor size, as was the case for EGFR mutation-positive NSCLC. The mean PFS of the patients in this study was 9.5 months, which was equivalent to that observed in patients with EGFR mutation-positive NSCLC (6–8). These results suggested that erlotinib may inhibit an unknown survival pathway or may act on tumors that have an unknown EGFR mutation status.

A number of limitations were present in the current study: i) The number of patients included was relatively small and, therefore, assessing the significance of differences was challenging and not necessarily representative of a larger population; ii) the retrospective nature of this study did not allow for a standardized measurement of PFS.

In conclusion, the findings suggest that the presence of pulmonary metastasis may be a predictive marker of the response to erlotinib in patients with EGFR mutation-negative NSCLC. Currently, EGFR mutation-negative NSCLC patients have been identified for whom treatment is terminated without receiving erlotinib. However, EGFR mutation-negative NSCLC patients with pulmonary metastasis may benefit from erlotinib treatment. A prospective clinical trial is required to confirm the efficacy of erlotinib treatment in EGFR mutation-negative NSCLC patients with pulmonary metastasis.

## Figures and Tables

**Table I:** Characteristics of the 53 non-small cell lung cancer patients.

Characteristics	Patients, n	%
Age, years		
Median	64	
Range	35–80	
Gender, n		
Male	40	75.5
Female	13	24.5
Smoking history, n		
Never	12	22.6
Former/current	41	77.4
Histology, n		
Adenocarcinoma	36	67.9
Squamous	13	24.5
Adeno-squamous/unidentified	1/3	1.9/5.7
Performance status, n		
0–1	44	83.0
2–3	9	17.0
Metastatic site, n		
Lung	27	50.9
Brain	13	24.5
Bone	11	20.8
Extrathoracic lymph node	10	18.9
Adrenal grand	5	9.4
Liver	6	11.3
Malignant pleural effusion	14	26.4
Others^a^	6	11.3

a Skin, 2; spleen, 1; muscle, 1; kidney, 1; peritoneum, 1.

**Table II:** RR, PFS and OS for the patients according to characteristics.

Factor	n	RR, %	P^a^	mPFS, mo	P-value^b^	mOS, mo	P-value^b^
Age, years							
>70	18	11.1	0.2493	1.9	0.1876	5.8	0.1151
<71	35	5.7		3.7		13.1	
Gender, n							
Male	40	5.0	0.2493	2.1	0.1235	5.8	0.1788
Female	13	15.4		3.9		16.6	
Smoking history, n							
Never	12	8.3	1.0000	2.3	0.2893	16.6	0.0975
Former/current	41	7.3		2.2		6.0	
Histology, n							
Adenocarcinoma	35	11.4	0.5619	1.8	0.2847	5.8	0.8179
Squamous	13	0.0		3.7		9.3	
Performance status, n							
0–1	44	9.1	1.0000	2.9	0.0002	8.6	<0.0001
2–3	9	0.0		0.5		1.9	
Skin rash, n							
Present	35	11.4	0.5619	2.9	0.0077	8.6	0.0026
Not present	18	0.0		1.0		2.8	

a Determined by Fisher’s exact test.

b Univariate analysis by log-rank test.

RR, response rate; mPFS, median progression-free survival; mOS, median overall survival; mo, months.

**Table III:** RR, PFS and OS for the 53 patients according to the presence of metastatic sites.

Metastatic site	n	RR, %	P-value^a^	mPFS, mo	P-value^b^	mOS, mo	P-value^b^
Pulmonary metastasis, n							
Yes	27	14.8	0.1110	2.9	0.0010	12.4	0.0007
No	26	0.0		1.2		4.1	
Brain metastasis, n							
Yes	13	7.7	1.0000	1.7	0.0440	5.0	0.0929
No	40	7.5		2.7		7.0	
Bone metastasis, n							
Yes	11	0.0	0.5688	1.2	0.0153	4.9	0.4427
No	42	9.5		2.7		7.0	
Extrathoracic lymph node metastasis, n							
Yes	10	0.0	1.0000	1.9	0.5291	7.0	0.3850
No	43	9.3		2.3		6.2	
Adrenal grand metastasis, n							
Yes	5	20.0	0.3355	1.7	0.3993	5.8	0.3109
No	48	6.3		2.5		7.0	
Liver metastasis, n							
Yes	6	0.0	1.0000	0.7	<0.0001	2.9	0.0004
No	47	8.5		2.5		7.6	
Malignant pleural effusion, n							
Yes	14	26.4	0.0034	2.1	0.4575	5.5	0.9935
No	39	0.0		2.5		7.3	

a Determined by Fisher’s exact test.

b Univariate analysis by log-rank test.

RR, response rate; mPFS, median progression-free survival; mOS, median overall survival; mo, months.

**Table IV:** Multivariate analysis of progression-free survival.

Independent factor	Hazard ratio	95% CI	P-value
Pulmonary metastasis	0.39	0.20–0.76	0.0055
Brain metastasis	0.94	0.40–2.06	0.8721
Bone metastasis	2.24	0.96–4.95	0.0616
Liver metastasis	3.82	1.17–11.65	0.0279
Onset of skin rash	0.49	0.25–1.01	0.0522
PS (2–3 vs. 0–1)	3.12	1.20–7.51	0.0214

Multivariate analysis by Cox proportional-hazards regression model. CI, confidence interval; PS, performance status.

**Table V:** Multivariate Analysis of overall survival.

Independent factor	Hazard ratio	95% CI	P-value
Pulmonary metastasis	0.33	0.16–0.67	0.0022
Liver metastasis	2.65	0.88–7.18	0.0801
Onset of skin rash	0.43	0.20–0.95	0.0381
PS (2–3 vs. 0–1)	3.74	1.36–9.84	0.0115

Multivariate analysis by Cox proportional-hazards regression model. CI, confidence interval; PS, performance status.
